# Dermatomyositis With Necrotizing Vasculitis, Pulmonary Arterial Hypertension, and Extensive Calcinosis Following Delayed Diagnosis

**DOI:** 10.7759/cureus.110687

**Published:** 2026-06-11

**Authors:** Amine Laabichi, Wafa Ammouri, Naima Mouatassim, Mouna Maamar, Hicham Harmouche

**Affiliations:** 1 Internal Medicine and Clinical Immunology, Centre Hospitalier Universitaire Ibn Sina, Rabat, MAR; 2 Acute Geriatrics Unit, Internal Medicine and Clinical Immunology, Centre Hospitalier Universitaire Ibn Sina, Rabat, MAR

**Keywords:** autoantibodies, calcinosis, inflammatory myopathy, pulmonary arterial hypertension, s: dermatomyositis, vasculitis

## Abstract

Dermatomyositis is a rare autoimmune inflammatory disease with a highly heterogeneous clinical presentation and potential for multisystem involvement. Early diagnosis may be challenging, particularly in patients presenting with amyopathic disease and limited initial laboratory abnormalities.

We report the case of a 45-year-old woman initially diagnosed with amyopathic dermatomyositis based on characteristic cutaneous manifestations in the absence of objective muscle involvement on laboratory testing and electromyography. After being lost to follow-up for four years, she presented with progressive proximal muscle weakness, extensive ulcerative calcinosis, Raynaud phenomenon, and exertional dyspnea. Extended myositis serology revealed anti-Mi-2 and anti-signal recognition particle (SRP) antibodies. Muscle biopsy demonstrated perifascicular atrophy, necrotizing vasculitis, and foreign-body granulomatous inflammation associated with dystrophic calcinosis. Right heart catheterization confirmed pre-capillary pulmonary arterial hypertension in the absence of interstitial lung disease.

The patient was treated with intravenous cyclophosphamide, pulmonary vasodilator therapy, colchicine, and tapering corticosteroids, resulting in clinical and functional improvement.

This case highlights the potential progression of initially amyopathic dermatomyositis to severe systemic disease, the limitations of isolated serological assessment, and the importance of long-term follow-up. It also illustrates several uncommon manifestations of adult dermatomyositis, including extensive calcinosis, pulmonary arterial hypertension, and histologically proven necrotizing vasculitis, supporting the concept of dermatomyositis as a systemic vasculopathic disorder.

## Introduction

Dermatomyositis is a rare autoimmune inflammatory disease characterized by distinctive cutaneous manifestations and variable degrees of muscle involvement. Although proximal muscle weakness is classically regarded as a hallmark feature, some patients initially present with isolated skin manifestations, making early diagnosis and assessment of disease severity more challenging. Beyond the skin and skeletal muscles, dermatomyositis is increasingly recognized as a systemic disease that may involve the lungs, cardiovascular system, and small vessels, resulting in substantial morbidity [1].

The identification of myositis-specific autoantibodies has considerably improved the diagnostic and prognostic evaluation of inflammatory myopathies. Certain autoantibodies are associated with characteristic clinical phenotypes and patterns of organ involvement. However, serological findings have important limitations: antibodies may be absent early in the disease course, present at low titres, or occur in combinations that do not correspond to established clinicopathological profiles. As a result, diagnosis and therapeutic decisions should remain guided by the overall clinical presentation rather than serology alone [2].

While cutaneous and muscular manifestations are common, severe calcinosis and pulmonary arterial hypertension (PAH) are uncommon complications in adult dermatomyositis. Calcinosis is more frequently encountered in juvenile disease, whereas PAH remains a rare but potentially life-threatening manifestation that may be overlooked because of its non-specific presentation. In addition, vascular involvement in dermatomyositis is increasingly recognized, although histologically proven necrotizing vasculitis remains exceptionally reported [3].

We report the case of a patient initially diagnosed with amyopathic dermatomyositis who was subsequently lost to follow-up and later presented with progressive systemic disease characterized by extensive ulcerative calcinosis, pre-capillary pulmonary arterial hypertension, necrotizing vasculitis, and an unusual autoantibody profile. This case highlights the limitations of early disease assessment, the consequences of delayed recognition and insufficient immunosuppression, and the importance of long-term multidisciplinary management in patients with inflammatory myopathies.

## Case presentation

A 45-year-old woman with no significant past medical history developed bilateral inflammatory polyarthralgia and characteristic dermatomyositis skin lesions (Gottron's papules and Gottron's sign, associated with Raynaud phenomenon) in late 2019. She also reported fatigue and unintentional weight loss. In early 2020, laboratory investigations, including antinuclear antibodies (ANA), antineutrophil cytoplasmic antibodies (ANCA), anti-cyclic citrullinated peptide antibodies (anti-CCP), rheumatoid factor, a limited myositis antibody panel, creatine kinase (CK), and electromyography, were all within normal limits. Amyopathic dermatomyositis was diagnosed based on the EULAR/ACR 2017 classification, with a score of 6.7 and an 80% probability.

She was treated with prednisone (1 mg/kg/day, tapered gradually) and methotrexate (20 mg/week), which was quickly discontinued due to a national shortage during the COVID-19 pandemic. Joint symptoms improved moderately, but the cutaneous lesions persisted. The patient was lost to follow-up from 2020 until 2024.

In December 2024, she presented again with progressive proximal muscle weakness (Medical Research Council grade 4/5), painful subcutaneous calcifications with overlying skin ulcerations (Figure 1) predominantly located at the proximal level of the limbs and over the large joints, as well as in the lower back and abdomen, Raynaud phenomenon, exertional dyspnea, diffuse hyperalgesia, and severe functional limitation (WHO performance status 3). Chronic corticosteroid exposure had led to bilateral cataracts, steroid-induced diabetes, and osteoporotic vertebral fractures at T12, L1, and L3.

**Figure 1:**
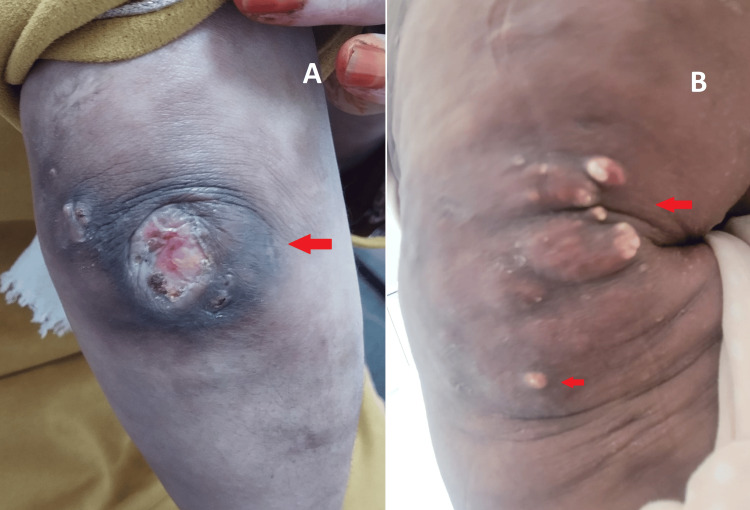
Cutaneous ulceration and exposed calcinosis On the left (A), a cutaneous ulceration at the elbow overlies subcutaneous calcinosis (red arrows); on the right (B), exposed calcinosis is visible on the thigh (red arrows).

On examination, she exhibited symmetrical proximal muscle weakness, spinal tenderness with a positive bell sign, and active ulcerative skin lesions. Laboratory tests showed a normal creatine kinase level (24 U/L), elevated C-reactive protein (35 mg/L), and normal calcium-phosphate levels. Standard radiography revealed multiple diffuse subcutaneous calcifications, especially in the proximal limbs and lower abdominal wall (Figure 2).

**Figure 2:**
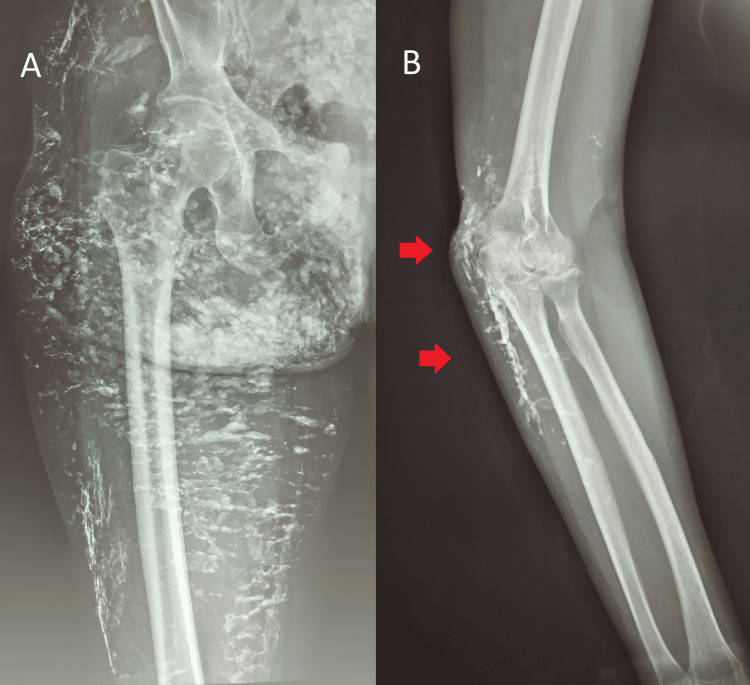
Radiographic evidence of subcutaneous calcinosis Radiographic image A shows extensive calcinosis of the thigh, while image B depicts subcutaneous calcifications around the elbows (red arrows).

An extended myositis antibody panel was positive for anti-Mi-2 (10 U) and anti-SRP (12 U) antibodies, with borderline anti-OJ (8 U). Muscle biopsy demonstrated myofiber atrophy, fibrinoid necrotizing vasculitis, and non-caseating epithelioid granulomas within subcutaneous fat (Figure 3).

**Figure 3:**
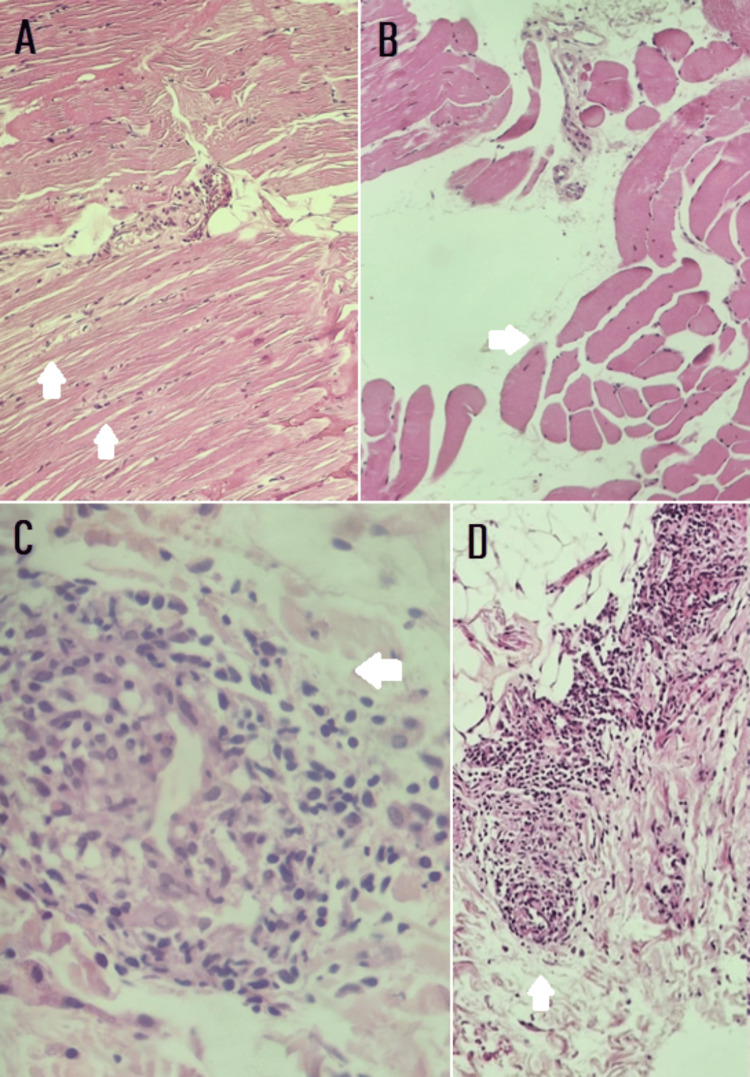
Histopathologic findings from the muscle biopsy illustrating inflammatory, granulomatous, and vascular involvement (A) Hematoxylin-eosin staining (HE), low magnification (×100), showing endomysial and perimysial inflammatory infiltrates (white arrows) with scattered necrotic muscle fibers. (B) HE staining, higher magnification (×200) revealing perifascicular muscle fiber atrophy (white arrow) and perivascular inflammation. (C) HE staining of the vascular wall in the perimysium (×400) surrounded by a dense polymorphic inflammatory infiltrate rich in neutrophils, disrupting the vascular basement membrane (white arrow), consistent with necrotizing vasculitis. (D) HE staining, well-formed non-caseating granuloma in subcutaneous adipose tissue (×200) (white arrow), composed of epithelioid histiocytes and multinucleated giant cells.

Sarcoidosis and tuberculosis were deemed unlikely on a normal angiotensin-converting enzyme (ACE) level (52 IU/L), negative sputum cultures for acid-fast bacilli, a negative tuberculin skin test, and unremarkable imaging studies. The histologic granulomas were therefore attributed to a foreign-body reaction secondary to dystrophic calcinosis.

Thoracic CT angiography (Figure 4) showed cardiomegaly, soft tissue calcifications, and signs of pulmonary hypertension without evidence of interstitial lung disease or mediastinal lymphadenopathy. Bone densitometry confirmed osteoporosis. Right heart catheterization demonstrated precapillary PAH, with a median pulmonary arterial pressure of 47 mmHg, a pulmonary vascular resistance of 9 Wood units, a pulmonary artery wedge pressure (PAWP) of 14 mmHg, a cardiac index of 2.7 L/min, and a mixed venous oxygen saturation of 56%.

**Figure 4:**
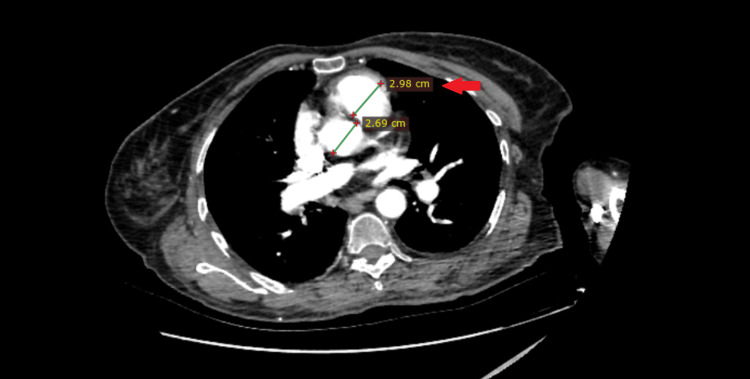
Axial chest CT showing main pulmonary artery dilatation CT: Computed tomography Contrast-enhanced axial CT image at the level of the main pulmonary artery. The artery measures 2.98 cm in diameter (red arrow), exceeding the upper limit of normal for females (29 mm). The ascending aorta measures 2.69 cm, yielding a pulmonary artery-to-aorta ratio (AP/Ao) of 1.11. These findings are consistent with pulmonary arterial hypertension later confirmed by heart catheterization.

Electromyography (EMG) was performed to investigate diffuse hyperalgesia and muscle-related symptoms. It revealed bilateral sensory axonal neuropathy. Given the patient's recent diagnosis of steroid-induced diabetes and absence of other diabetic complications (retinopathy, nephropathy), this finding was initially attributed to diabetes, but its etiology remains uncertain.

Toward the end of the diagnostic work-up, a PET-CT scan was performed to screen for occult malignancy. While no suspicious neoplastic lesions were identified, the scan revealed intense fluorodeoxyglucose (FDG) uptake in the calcified subcutaneous masses in both thighs (Figure 5). This finding indicated metabolically active inflammation within the calcifications, a pattern later confirmed by histology, which showed a foreign-body granulomatous reaction in the subcutaneous fat tissue.

**Figure 5:**
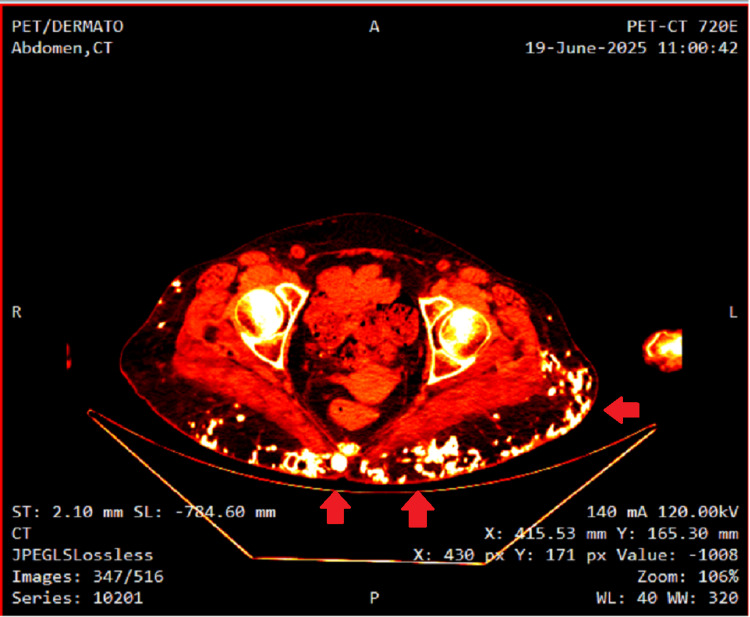
PET/CT showing metabolically active calcinosis PET: Positron emission tomography, CT: Computed tomography Fused PET/CT image demonstrating intense FDG uptake within subcutaneous calcified masses in both thighs (SUV max = 4.5), consistent with metabolically active calcinosis (red arrows). The pattern of uptake supported the diagnosis of an inflammatory foreign-body reaction that was confirmed on histology.

The patient was started on monthly intravenous cyclophosphamide (1,000 mg), with plans to transition to maintenance therapy with mycophenolate mofetil after six cycles. Macitentan and tadalafil were introduced for PAH, and colchicine was prescribed for calcinosis. The patient responded well to treatment, with marked improvement in muscle strength (5/5 after the first month) and reduction in pain (2/10 on the Numerical Rating Scale versus 6/10 at admission). Follow-up echocardiography at three months and radiographic imaging of calcinosis at six months were scheduled.

## Discussion

This case illustrates the heterogeneous and potentially progressive nature of dermatomyositis. The patient initially fulfilled criteria for amyopathic dermatomyositis, presenting with characteristic cutaneous manifestations in the absence of objective muscle involvement on laboratory testing and electromyography. However, over the following years, the disease evolved toward a multisystem phenotype characterized by proximal muscle weakness, extensive calcinosis, pulmonary arterial hypertension (PAH), and histologically documented necrotizing vasculitis. This evolution highlights the limitations of a single baseline assessment and underscores the importance of long-term follow-up in patients with inflammatory myopathies, even when the initial presentation appears limited.

Another important lesson from this case concerns the consequences of delayed immunosuppressive therapy. Following the interruption of methotrexate and prolonged exposure to corticosteroid monotherapy, the patient developed severe disease-related and treatment-related complications, including calcinosis, osteoporosis, vertebral fractures, diabetes mellitus, and cataracts. This observation supports current recommendations favoring early introduction of steroid-sparing immunosuppressive agents whenever possible and highlights the substantial morbidity associated with prolonged corticosteroid exposure [4].

Myositis-specific autoantibodies have considerably improved the characterization of inflammatory myopathies and are increasingly used to predict disease phenotype and prognosis. Nevertheless, serology should be interpreted within the broader clinical and pathological context. The simultaneous presence of more than one myositis-specific antibody is uncommon but increasingly recognized. A recent case series by Zhang et al. [5] described patients with co-expression of anti-SRP and another myositis-specific antibody, demonstrating marked clinical heterogeneity. The authors observed that one antibody often appears to dominate the clinical phenotype. In our patients, despite the coexistence of anti-Mi-2 and anti-SRP antibodies, the overall presentation, including classic dermatomyositis skin lesions, perifascicular atrophy, and extensive calcinosis, was more consistent with dermatomyositis than with immune-mediated necrotizing myopathy. This case, therefore, illustrates both the value and limitations of serological profiling and reinforces the need for phenotype-driven interpretation of antibody results.

The extent and severity of calcinosis observed in our patient are unusual for adult dermatomyositis and are more commonly reported in juvenile disease [6]. Beyond causing pain and functional impairment, the calcified lesions became ulcerated and were associated with a granulomatous foreign-body reaction. Management of calcinosis remains challenging, with limited therapeutic options and variable responses to available treatments. The severity of calcinosis in this patient may reflect prolonged uncontrolled inflammation resulting from delayed disease control.

Dermatomyositis is increasingly recognized as a systemic vasculopathic disorder rather than a disease confined to the skin and skeletal muscles. Vascular injury is believed to play a central role in disease pathogenesis through complement-mediated endothelial damage, capillary loss, and microangiopathy. Severe vasculopathic manifestations have been associated with cutaneous ulceration, calcinosis, and extra-muscular organ involvement [3]. In this context, the coexistence of ulcerative calcinosis, pulmonary arterial hypertension, and histologically proven necrotizing vasculitis in our patient raises the possibility of a predominantly vasculopathic disease phenotype. Although a direct pathogenic relationship cannot be established, these findings support the concept that vascular injury may contribute substantially to disease severity in selected patients.

Emerging evidence suggests that neuromyositis, the coexistence of inflammatory myopathy and peripheral neuropathy, is a rare but recognized phenomenon. Senator H. first described this association in the late nineteenth century [7], and subsequent reports have documented patients presenting with both muscle and nerve involvement [8,9]. In our patient, electromyography demonstrated bilateral sensory axonal neuropathy. Although initially attributed to recently diagnosed diabetes mellitus, the absence of other diabetic microvascular complications and the concurrent finding of necrotizing vasculitis raise the possibility of a vasculitic neuropathy related to the underlying autoimmune disease. The clinical response of the neuropathy to immunosuppressive therapy may provide indirect support for this hypothesis during follow-up.

PAH in the setting of idiopathic inflammatory myopathies is uncommon but increasingly recognized. A French registry study identified three cases of PAH among 34 patients with inflammatory myopathies without extensive interstitial lung disease, all of whom had dermatomyositis [10]. These patients exhibited hemodynamic characteristics similar to those observed in systemic sclerosis-associated PAH and responded favorably to pulmonary vasodilator therapy. Our patient's profile, pre-capillary PAH (PVR 9 WU, PAWP <15 mmHg) in the absence of interstitial lung disease, closely mirrors these observations and is consistent with ESC Group 1.4.1 pulmonary arterial hypertension associated with connective tissue disease. This case supports maintaining a high index of suspicion for PAH in dermatomyositis patients presenting with unexplained dyspnea, even in the absence of interstitial lung disease.

Interestingly, the calcified lesions demonstrated intense FDG uptake on PET-CT imaging, initially raising concern for malignancy or infection. Similar findings have occasionally been reported in inflammatory calcinosis and foreign-body granulomatous reactions [11]. In our patient, histology demonstrated non-caseating granulomas associated with dystrophic calcinosis, supporting an inflammatory foreign-body response rather than neoplasia. This observation emphasizes the importance of correlating PET findings with histopathology, as increased metabolic activity within calcified lesions does not necessarily indicate malignancy. PET-CT may therefore represent a useful adjunctive tool in selected complex cases, although its role in dermatomyositis remains to be further defined.

## Conclusions

This case highlights the heterogeneous presentation and potentially severe evolution of dermatomyositis. What initially appeared to be amyopathic disease progressed to a multisystem disorder characterized by extensive calcinosis, pulmonary arterial hypertension, sensory neuropathy, and biopsy-proven necrotizing vasculitis. Beyond illustrating the limitations of early serological assessment, this observation supports the concept of dermatomyositis as a systemic vasculopathic disease in which vascular injury may contribute to both muscular and extra-muscular manifestations.

Recognition of atypical or evolving phenotypes is crucial, as delayed diagnosis and insufficient immunosuppression may result in irreversible disease-related and treatment-related complications. Comprehensive assessment and multidisciplinary management remain key to improving long-term outcomes in patients with dermatomyositis.

## References

[1] Allenbach Y, Benveniste O (2026). Inflammatory myopathies. N Engl J Med.

[2] Betteridge Z, McHugh N (2016). Myositis-specific autoantibodies: an important tool to support diagnosis of myositis. J Intern Med.

[3] Mammen AL, Allenbach Y, Stenzel W, Benveniste O (2020). 239th ENMC International Workshop: Classification of dermatomyositis, Amsterdam, the Netherlands, 14-16 December 2018. Neuromuscul Disord.

[4] Campanilho-Marques R, Fonseca JE, Machado PM (2025). Treatment of idiopathic inflammatory myopathies. Joint Bone Spine.

[5] Zheng Y, Zhao Y, Hao H (2023). What should we expect when two myositis-specific antibodies coexist in a patient. Eur J Med Res.

[6] Mathiesen P, Hegaard H, Herlin T (2012). Long-term outcome in patients with juvenile dermatomyositis: a cross-sectional follow-up study. Scand J Rheumatol.

[7] Senator Hermann (1893). Ueber acute polymyositis und neuromyositis. Dtsch Med Wochenschr.

[8] Lei-Ying Huang, Yi-Jen Peng, Yueh-Feng Sung (2023). Anti-SRP myopathy with sensorimotor polyneuropathy: a case report. Acta Neurol Taiwan.

[9] Kumari S, Priyanka G, N C K, Bansal R, Sharma S (2025). Converging inflammations: simultaneous myositis and polyneuropathy as a diagnostic challenge. Cureus.

[10] Sanges S, Yelnik CM, Sitbon O (2016). Pulmonary arterial hypertension in idiopathic inflammatory myopathies: data from the French pulmonary hypertension registry and review of the literature. Medicine (Baltimore).

[11] Miyamae T, Sano F, Ozawa R (2010). Efficacy of thalidomide in a girl with inflammatory calcinosis, a severe complication of juvenile dermatomyositis. Pediatr Rheumatol Online J.

